# Weight Loss Motivation in Older Pre-Diabetics: Preliminary Evidence by Race in the Eggspdite Study

**DOI:** 10.1093/geroni/igaa057.739

**Published:** 2020-12-16

**Authors:** Marshall Miller, Michael Borack, Jamie Rincker, Shelley McDonald, Kathyrn Starr, Connie Bales

**Affiliations:** 1 Duke University, Efland, North Carolina, United States; 2 Duke University, Durham, North Carolina, United States; 3 Duke University School of Medicine, Durham, North Carolina, United States

## Abstract

Obesity hastens functional decline and intensifies chronic health conditions among older adults. Late-life obesity is of particular concern for older African Americans, who are at increased risk for obesity and type 2 diabetes and for whom weight loss interventions can be less effective. However, obesity interventions have been under-studied in this population; little is known about potential differences in motivation for change by race. The ongoing Eggs PreDiabetes Intervention Trial (EGGSPDITe) is a randomized controlled trial of expedited weight loss in older (60+ years) Black and White adults with prediabetes. Participants completed both the Stages- and Processes of Change questionnaires in Weight Management (S-Weight and P-Weight) at baseline and end of 4-month intervention. Preliminary combined-group analysis indicates that, while White participants reported a higher average stage of change at baseline, there was no difference by race (ps < 0.05) in changes for body weight, fat mass, and hemoglobin A1c at endpoint. Reductions in weight consequences evaluation (WCE) and increases in weight management actions (WMA) subscales were observed in both races (ps < 0.05), with a trend toward decreased emotional re-evaluation (EmR; p = 0.06). Of the processes of change subscales, only WCE differed by race (p < 0.05), with White participants showing ≈13.5% higher utilization, relative to Black participants, at both time points. These preliminary findings suggest that weight loss interventions can be equally effective among black and white older adults, although motivations for weight loss may differ by race.

